# The biology and treatment of oligometastatic cancer

**DOI:** 10.18632/oncotarget.3455

**Published:** 2015-04-13

**Authors:** Diane K. Reyes, Kenneth J. Pienta

**Affiliations:** ^1^ Departments of Urology and Brady Urological Institute, and Oncology, The Johns Hopkins Medical Institutions, Baltimore, MD, 21287, USA; ^2^ Departments of Pharmacology and Molecular Sciences, and Chemical and Biomolecular Engineering, The Johns Hopkins Medical Institutions, Baltimore, MD, 21287, USA

**Keywords:** metastasis, therapy, tumor, spectrum theory, diaspora

## Abstract

Clinical reports of limited and treatable cancer metastases, a disease state that exists in a transitional zone between localized and widespread systemic disease, were noted on occasion historically and are now termed oligometastasis. The ramification of a diagnosis of oligometastasis is a change in treatment paradigm, i.e. if the primary cancer site (if still present) is controlled, or resected, and the metastatic sites are ablated (surgically or with radiation), a prolonged disease-free interval, and perhaps even cure, may be achieved. Contemporary molecular diagnostics are edging closer to being able to determine where an individual metastatic deposit is within the continuum of malignancy. Preclinical models are on the outset of laying the groundwork for understanding the oligometastatic state. Meanwhile, in the clinic, patients are increasingly being designated as having oligometastatic disease and being treated owing to improved diagnostic imaging, novel treatment options with the potential to provide either direct or bridging therapy, and progressively broad definitions of oligometastasis.

## INTRODUCTION

Hellman first proposed the theory of oligometastases in 1995 as a sequel to the spectrum theory of cancer metastasis. Hellman hypothesized that the process of cancer metastases occurred along a continuum, from locally confined cancer to widely metastatic disease. Although the phenomenon of limited and treatable cancer metastases had been noted historically, Hellman and Weichselbaum proposed the term oligometastases, suggesting that in some patients with a limited number of clinically detectable metastatic tumors, the extent of disease exists in a transitional state between localized and widespread systemic disease. In this model local control (LC) of oligometastases would have the potential to yield improved systemic control, going against the dogma that control of oligometastatic disease would not have a therapeutic benefit since it represents a clinical manifestation of a few detectable lesions in the setting of widespread occult disease. Oligometastasis continues to be defined as a state of metastatic disease that is limited in total disease burden, usually by number of clinically evident or radiographic sites (either 1–3 or 1–5), and that is not rapidly spreading to more sites. The clinical implication of oligometastasis suggests that if the primary site (if still present) is controlled, or resected, and the metastatic sites are ablated (surgically or with radiation), there will be a prolonged disease-free interval, and perhaps even cure. As the understanding of the mechanisms underlying cancer metastasis have evolved, possible mechanisms for the oligometastatic state must be explained and examined within that context.

### Biological basis for oligometastasis

#### Theories of metastasis

Metastasis is the cause of most cancer-related deaths [1]. In 1889, Stephen Paget [2] theorized that circulating tumor cells would “seed” to an amenable “soil”, suggesting that metastasis was not a matter of chance. Five years later, [3, 4], in 1894, Halstead theorized that cancer was an orderly disease that progressed in a contiguous manner, by direct extension from the primary tumor through the lymphatics, to the lymph nodes, and then to distant sites. Halstead proposed that breast cancer metastasis was a progressive, anatomical process of contiguous seeding; his hypothesis supported the use of radical surgery and radiotherapy. Radical en bloc surgery, radical hysterectomy, and primary and regional irradiation for several tumor sites were all based on Halstead's theory of tumor spread [4]. James Ewing, in 1928, complemented the Paget and Halstead theories to propose that cancer cells grow at a particular site because they are directed by the direction of blood flow and lymphatics. [5]

The ‘systemic’ theory of metastasis, first suggested by Keynes [6] and further developed by Fisher [7], held that clinically apparent cancer was a systemic disease, and that small tumors were an early manifestation of systemic disease [8]. In this theory, nodal involvement was not part of an orderly contiguous extension but rather a marker of distant metastases. According to this theory, local control would not impact survival.

In contrast to the ‘Halsted’ theory and the ‘systemic’ theory, the ‘spectrum’ theory of cancer metastases, first described for breast cancer metastases in 1994, held that disease stage at the time of initial disease presentation fell into a spectrum ranging from indolent disease to widely metastatic, with the degree of clonal evolution determining the ability of the tumor to metastasize [9]. The spectrum theory was refined just one year later to describe the limited metastasis of any solid tumor and the term ‘oligometastasis’ was coined.

**Figure 1:**
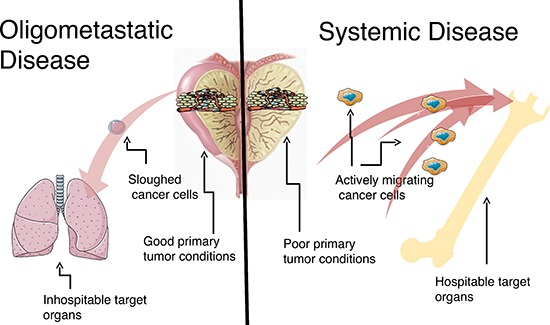
Oligometastatic disease *versus* systemic disease (left) Oligometastatic disease. Metastatic growth potential is limited. This could be (a) secondary to due to environmental conditions in the primary tumor forestalling evolutionary clonal pressure, (b) cancer cells that slough out of the primary tumor that do not have the properties necessary to survive the circulation and invade into target organ sites, and/or (c) the cancer cells land in inhospitable target organs. (right) Systemic disease. Widespread metastatic growth potential is unlimited. This could be (a) secondary to due to environmental conditions in the primary tumor creating many undifferentiated, aggressive clones, (b) cancer cells that actively migrate out of the primary tumor that have the properties necessary to survive the circulation and invade into target organ sites, and/or (c) the cancer cells land in hospitable target organs.

The spectrum theory conceptualized the entire range of metastatic competence, analogous to a diapason, which is the entire range of an instrument. To that end, the social sciences concept of a diaspora has recently been utilized to inform biologic understanding and therapeutic paradigms of cancer metastasis [10]. A diaspora refers to the scattering or movement of a population from its original homeland. In the case of systemic metastases, the diaspora resembles an imperial colonization in which the populations spread widely and eventually conquer the new host lands (aggressive cancer clones to multiple organs). Oligometastases resemble trading post diasporas, representing a limited number of outposts with limited growth potential (less aggressive cancer clones to few organs). (Table 1, Figure 1). Systemic versus oligometastatic diasporas may be dependent on the types of mutations present in the cancer cells (quality of the diaspora migrants), the quality of the original tumor site (factors in the homeland that cause the population to migrate), and the quality of the new hostland (factors that allow immigrants to establish and flourish) (Table 2).

**Table 1:** A comparison of migrants, diaspora, and *the spectrum* of cancer metastases

Social Demography		Cancer Demography	
Imperial Diaspora	Trading Post Diaspora	*Trading Post Diaspora → Oligometastasis*	*Imperial Diaspora → Cancer metastasis*
Large populations from a single homeland	Small population from a single homeland	Migrated from primary cancer in passive manner	Dispersed from a primary cancer in an active manner
Settle multiple countries in aggressive manner	Settle in few countries while avoiding upsetting host country	Mild hypoxia and unlimited nutrients; Home niche conditions do not cause evolutionary clonal pressure	Hypoxia and lack of nutrients cause pressure to leave primary; Evolving home niche conditions cause undifferentiated, aggressive clones.
Host country may or may not be receptive	Host country may or may not be receptive	Target organ may or may not be receptive	Target organ may or may not be receptive
Group maintains collective memory of their homeland and culture	Group maintains collective memory of their homeland and culture	Pathologists can identify where a cancer cell originated	Pathologists can identify where a cancer cell originated
Often assimilate the new homeland	Survive as distinct communities	Few distinct metastases	Multiple metastases as distinct masses
Relationship with host country is uneasy and degenerates over time	Relationship with host country may be uneasy but is maintained over time	Immune system may not see a threat	Immune system tries to destroy the cancer cells
Tied to the homeland by exchange of resources	Tied to the homeland by exchange of resources	Limited need for outside resources from homeland; fewer cells trafficking	Multiple cell-type trafficking, trafficking of resources/info

Table adapted from Pienta et al. Clin Can Research, 2013 [10]

**Table 2:** Factors that define the rate and success or failure of oligometastases and systemic metastases

**Cancer dispersion**	**Quality of primary microenvironment [_Δ_Q_O_][_Δ_t]**	**Fitness of migrant cancer cells (E_FnΔt_) + (M_FnΔt_) + (S_FnΔt_)**	**Quality of metastatic sites (H1_QnΔt)_) + (H2_QnΔt)_)…**
**Oligometastasis**	**_Δ_Q_O_ = little change**	**[(E_FnΔt_) > (M_FnΔt_) (S_FnΔt_)] [TR]**	**[(H1_Qn)_) + (H2_Qn)_] = low**
**Widespread metastasis**	**_Δ_Q_O_ = decreasing**	**[(E_FnΔt_) < (M_FnΔt_) (S_FnΔt_)] [TR]**	**[(H1_Qn)_) + (H2_Qn)_) = high**
**Primary microenvironment quality:**_Δ_*Q_O_* = the changing quality (_Δ_*Q*) of the primary cancer site over time (_Δ_t). The quality of the primary microenvironment is dependent on multiple factors, including pH, oxygenation, amount of nutrients, interaction with supporting host cells, and the quality of the immune response. Primary nonlethal, epithelial cancer cells in a highly vascularized environment with rich nutrients are presumed to be less likely to evolve to a more aggressive clone by undergoing an epithelial to mesenchymal transition (EMT) and leave the primary over time. As the quality of the environment decreases, it is likely that the generation of lethal, mesenchymal clones increases.			
**Fitness of migrant cancer cells:** E_*Fn*Δ*t*_ = the number (*n*) and fitness (*F*) of passively shed epithelial cancer cells (E) over time (Δ*t*). This represents the likelihood that a cancer cell passively shed into the circulation will survive transport to a target organ. M_*Fn*Δ*t*_ = the number (*n*) and fitness (*F*) of actively emigrant cancer cells (M) over time (Δ*t*). S_FnΔt_ = number (*n*) and fitness (*F*) of cancer stem cells (S) over time. It is likely that the fitness of a passively shed cell is less than a migrating mesenchymal cell or stem cell that actively exits the primary tumor through the lymphatics, nerves, or circulation. Fitness depends on many variables, including EMT state, stemness, ability to secrete MMPs, ability to avoid anoikis, etc.			
**Quality of metastatic sites:** H*_Qn_* = the quality (*Q*) of the target organ or host-land sites (H1, H2…). Migrating cancer cells will land in multiple sites (*n*) within different target organs in order to immigrate. Success depends on the quality of the soil of each of these microenvironments as well as the host immune response.			

Table adapted from Pienta et al. Clin Can Research, 2013, [10]

### Biology of systemic and oligometastases

It is now widely accepted that there are discrete steps in tumor metastasis. Initially there is a loss in cellular adhesion, followed by increased motility > invasiveness of the primary tumor > entry into and survival in the circulation > entry into new organs > eventual colonization of these organs [11, 12]. Shortfalls at any stage of this metastatic progression could result in phenotypes of limited metastatic potential [13]. Gupta et al. described specific tumorigenic genes – initiation, progression, and virulence genes – that fulfilled specific roles in the metastatic cascade. Initiation genes afford a selective advantage to primary tumor cells to enter circulation. Progression genes fulfill rate-limiting steps for colonization. Virulence genes provide a selective advantage in cells to colonize a secondary site(s) [14]. As Weichselbaum and Hellman noted, this paradigm suggests “that there may be primary tumor cells with a limited capability in one or more of the necessary biological requirements for metastasis; thus proposing a possible biologic explanation of the origin of oligometastases” [15].

In 2000 and updated in 2011, Hanahan and Weinberg proposed the, widely accepted, ‘hallmarks of cancer’ [16]. The original cancer hallmarks consisted of six transformations in cellular physiology that allow cells to survive, proliferate and disseminate, and together support carcinogenesis. The update posited that underlying the original hallmarks were two ‘enabling hallmarks’: ‘genome instability and mutation’ and ‘tumor-promoting inflammation’. Additionally, the update proposed two new ‘emerging hallmarks’: ‘deregulation of cellular energetics’ and ‘avoidance of immune destruction’ [17]. The exact sequence in which the transformations occur, and thus the appearance of the ‘hallmarks’ (self-sufficiency in growth signals ↔ insensitivity to anti-growth signals ↔ tissue evasion and metastasis ↔ limitless replicative potential ↔ sustained angiogenesis ↔ evading apoptosis) can vary throughout the course of progression. Despite the ordering, collectively, the hallmarks can terminate into a cancer. However, the ordering and the degree of specific hallmarks, may potentially allow for a subsequent oligometastatic state. For example, cancer cells that lack the hallmarks to actively metastasize still may be able to slough into the circulation and inefficiently establish metastases. Other cells may be less efficient at proliferation, establishing slow growing metastases. Warburg (1956) suggested that “We may have cells which indeed look like cancer cells but are still energetically insufficient… such cells which are clinically not cancer cells, have lately been found not only in the prostate, but also in the lungs, kidney, and stomach of elderly persons such cells have been referred to as “sleeping cancer cells” [18].

### Preclinical models of oligometastasis

Traditional clinicopathologic factors are inadequate when attempting to define the potential underlying biology of oligometastases. Several investigations have demonstrated the marked genetic and epigenetic heterogeneity present in metastatic cancer sites within the same patient [19–22]. These studies demonstrate that cancer cells at different sites within a patient can have varied malignant potential [23–25]. Preclinical models of tumors with varying degrees of metastatic potential, including low metastatic potential exist. Using a cell line derived from B16F1 melanoma, Fidler et al. [26] found that variant metastatic cells pre-exist in a heterogeneous primary tumor as opposed to originating through adaption during metastasis from an otherwise homogenous primary tumor. This finding was advanced in later work that showed the KHT sarcoma line demonstrated similar heterogeneity whether grown in vitro or in vivo, which suggested that clonal variation seen in vitro derived from heterogeneity present in the primary tumor [27]. As an extension of this work in the KHT sarcoma line, it was demonstrated that effective metastatic variants developed at a high rate with low frequency, as opposed to the more frequent and stable subpopulations of metastatic variants [28]. In comparing B16 cell lines, Cillo et al. showed that the more highly metastatic, and less genetically stable cell line, generated increased metastatic variants corresponding to increased chemotherapy resistance [29]. Numerous *in vitro* studies and analyses of animal models have indicated that cells isolated from metastases differ greatly—both genetically and phenotypically—from cells isolated from their parental primary tumors [30]. The preclinical models point toward variation in individual tumor cells' metastatic potential, which supports the concept of oligometastases [15].

Given that stochastic models have been used to predict biologic phenomena, a Bayesian model has been proposed to predict the chance of occult metastases in the presence of detectable oligometastases [31]. Using the size and number of metastases, the proposed model inferred, (1) that the probability of occult metastases may increase substantially with minor increases in metastatic potential and (2) that extended disease-free periods were predictive of a substantial decrease in additional occult disease. Although compelling, such models are in their infancy and as yet remain in pre-clinical testing where the host, tumor and experimental factors are controlled. [31]

### Clinical evidence of oligometastasis

Evidence for the evolution of the oligometastatic phenotype comes from various clinical and pre-clinical sources [26, 32–34]. Recent studies of the molecular biology of renal cell cancer metastasis have implied biologic differences between less and more aggressive metastases, as well as between fewer and multiple metastases. In order to better determine which patients presenting with localized RCC harbor an aggressive tumor and may not benefit from surgery, Kosari et al. using gene expression profiling, found gene expression alterations associated with an aggressive tumor and metastatic potential in the primary tumor [35]. From a cohort of 20 resected pulmonary metastases taken from 18 patients, Wuttig et al demonstrated the predictive potential of identified gene signatures, when comparing disease-free intervals (DFI) and number of metastases, both of which are predictive of prognosis in metastatic RCC (mRCC). There were 306 differentially expressed genes in comparing DFI ≥5 years and DFI ≤9 months, and 135 differentially expressed genes in comparing multiple metastases (≥16) and few metastases (≤8). [36]

In colorectal cancer, there is growing evidence that liver-limited disease is a distinct biological cohort that may benefit from aggressive management. While only a minority of patients are technically resectable, approximately 40% of patients with resected liver limited disease are alive 5 years after diagnosis compared with less than 1% for those with disseminated disease. [37] There is genetic evidence that patients undergoing hepatic resection for metastatic cancer had a different disease than those who did not [37]. For example, it was noted that BRAF V600E mutant tumors, which were typically associated with aggressive biology, rarely came to liver resection [38, 39]. In addition, novel chromosomal aberrations have been identified that are associated with intra- and extra-hepatic recurrence after liver resection [40].

MicroRNAs, small non-coding RNA known to regulate tumor proliferation and apoptosis, are frequently dysregulated in cancer and metastasis [41–43]. MicroRNA profiling has shown a possible method to distinguish patients with oligometastases from those with polymetastatic disease. Examples of pro-metastatic microRNAs include microRNA-10b (upregulated in primary breast tumors that had metastasized), microRNA-21 (correlated with advanced stage, incidence of metastases, and poor outcomes in breast and pancreatic tumors), and microRNA-373/520c (increased expression in breast metastases) [43]. MicroRNA-210, a known transcriptional target of the HIF-1α signaling pathway, was elevated in sera from patients with metastatic castrate-resistant prostate cancer, as compared to controls, and was correlated with treatment response as assessed by change in PSA [41]. Lussiter et al. found microRNA-200c was associated with polymetastatic progression in a oligometastatic cell line, derived from patients treated with high-dose radiotherapy, tested in a xenograft model [44]. The investigators then stratified patients with resected pulmonary oligometastases into subgroups, based on high-risk versus low-risk of further metastatic progression. Differential microRNA expression patterns were identified between these two groups (high rate of progression (*n* = 16 prioritized microRNAs) and low rate of progression (*n* = 32 prioritized microRNAs) and, in an independent dataset, the expression patterns were associated with risk of progression and decreased overall survival. [8] Most recently, Uppal et al. identified three microRNAs overexpressed in clinical metastasis samples from patients with limited metastatic disease. MicroRNA-127–5p, microRNA-544a, and microRNA-655–3p were shown to limit, but not fully inhibit, metastasis in a model of breast cancer lung colonization [45].

### Controversies surrounding the treatment of oligometastatic disease

Hellman theorized that, 1) whereas some tumors were destined to remain localized, 2) other tumors, as they increased in size, acquired an increasingly greater metastatic phenotype, suggesting at an early stage these tumors seeded distant sites with clones that had not reached full metastatic potential, and finally, 3) that some tumors already had occult distant dissemination at the time they are diagnosed. He also proposed that metastatic potential was not only directed by the tumor phenotype, but that it was also influenced by the tumor's location, venous drainage, and host factors [4, 46]. Based on Hellman's theory, later scientists further theorized that tumors in the oligometastatic disease state were tumors early in their evolution of metastatic progression; therefore they produced metastases that were limited in number and location. These data support the presumption of a temporal evolution with an intermediate stage of limited metastatic capacity, where oligometastatic tumors may not have acquired the broad array of genetic changes required to develop widespread metastases [14, 47, 48].

The clinical implication of the oligometastatic state is that locally ablative therapies, given with the intent of targeting sites of clinically evident metastatic disease could result in long-term survival or cure [15, 49]. Treatment of oligometastatic disease may also result in decreased overall tumor burden, decreasing morbidity and increasing survival. These arguments are opposed by the concept that clinical metastases are evidence of systemic disease and locally directed treatment will not alter the natural history of the disease course within a patient. In this scenario, only systemic therapy may be beneficial. Indeed, oligometastatic treatment paradigms are controversial (due to the limited data available) [4, 34, 50, 51]. Without randomized studies, it is impossible to know if treatment of oligometastatic disease helps the patient. In addition, oligometastatic disease may represent indolent disease that does not require potentially toxic treatments [4, 34, 50, 51].

Patients are increasingly being diagnosed with oligometastatic disease due to the advent of sensitive imaging technologies as well as effective therapies that are allowing patients to live longer with the diagnosis of cancer [34, 52]. In addition, the fact that novel treatment options with acceptable safety profiles, such as stereotactic radiation, cryoablation, and minimally invasive surgery, are available to treat limited metastases, has led to a renewed interest in treating oligometastatic disease. Treatment of oligometastatic disease not only has the potential to prevent further evolution of genetically unstable clones and metastatic spread, it may improve overall disease control and delay more toxic systemic treatment [13, 34, 53, 54]. Finally, the definition of oligometastases had gradually evolved (Table 3), which further inflates the increasing population of patients diagnosed with oligometastasis. In the absence of data to guide decisions, treatment of oligometastatic disease may be seen as a quality-of-life oriented approach, choosing personalized treatments with a reasonable risk to benefit ratio and taking into account the patient's own attitude in guiding them toward more or less, intensive therapy [50].

**Table 3:** Definitions of Oligometastasis

Terms	Definition	Reference
Oligometastasis	“…metastases (from tumors early in the chain of progression) limited in number and location because the facility for metastatic growth has not been fully developed and the site for growth is restricted…”	[46]
Oligometastatic disease	Solitary or few detectable metastatic lesions that are usually confined to a single organ	[50]
Oligometastases	Due to limited metastatic competence and does not occur following otherwise successful systemic treatment. New metastases in this situation, albeit even limited, is likely to have more extensive malignant capabilities that were somehow spared from eradication by therapeutic means, or from the development of resistant clones	[15]
Induced oligometastases	Occurs when widespread micrometastatic disease is mostly eradicated by systemic chemotherapy but drug resistant clones are left behind, or tumor foci is located in a site not accessed by chemotherapy	[4]
Oligorecurrence	Limited metastases in the presence of a controlled primary lesion	[195]
Sync-oligometastases	≤5 metastatic or recurrent lesions in the presence of active primary lesions	[196]
Synchronous oligometastasis	Oligometastatic disease is detected at the time of diagnosis of the primary tumor, therefore there is an active primary tumor	[196]
Metachronous oligometastasis	Development of oligometastatic disease after treatment of the primary tumor; interval for classification of metachronous versus synchronous is not standardized; between Controlled primary lesion except for concomitant primary and distant recurrence	[196]
Oligoprogression	Progression of a limited number of metastatic deposits, while remaining metastases are controlled with systemic therapy	[197]
Oligometastasis (specific to prostate cancer)	Rising PSA following primary therapy, with oligometastasis on imaging, in whom local treatment (surgical metastasectomy (usually LN dissection), or SBRT for bony mets or LN recurrence) is required to defer initiation of ADT	[54]
Oligometastasis (specific to prostate cancer)	Castrate resistant prostate cancer with a rising PSA and oligometastasis on imaging, in whom local treatment (surgical metastasectomy (usually LN dissection), or SBRT for bony mets or LN recurrence) may allow deferral of ADT	[54]

Abbreviations: LN = lymph node; SBRT = stereotactic body radiation therapy; mets = metastases; ADT = androgen deprivation therapy; PSA = prostate-specific antigen

### Therapeutic options for oligometastases

#### Radiation therapy

While much of the literature supporting the oligometastatic states is within the surgical literature, there is an increasing body of literature describing the use of stereotactic body radiotherapy (SBRT) and stereotactic radiosurgery (SRS), in addition to conventional fractionated radiotherapy techniques [51]. SBRT is a noninvasive method of delivering high doses of radiation to ablate a target lesion while sparing the neighboring normal tissue, thus reducing long-term effects of radiation on the non-malignant tissues. The radiation is delivered from many beams originating from multiple directions that converge on the target site. [55] Through improved targeting and management of tumor motion, SBRT may improve tumor control and reduce treatment-related toxicity, as compared to conventional fractionated RT. Improved radiation targeting allows for higher-dose, hypofractionated, more efficient treatment regimens that can be delivered within narrow margins sparring adjacent organs. [56] ‘Hypofractionation’ is the delivery of large doses of radiation over a shorter time period as compared to conventional radiation fraction sizes. Therapy can generally be completed in 1–5 sessions, as compared to conventional radiation therapy that is delivered in smaller doses 5 days/week over ≥ 6 weeks. [55]

SBRT can be used to manage oligometastatic disease presentations that would be associated with added morbidity if managed by surgery, such as deep-seated or osseous lesions. Patients who are poor surgical candidates may often be treated with SBRT, given that it is noninvasive and has a modest morbidity profile. Whereas surgery, when used to manage oligometastatic disease, tends to be seen as the gold standard as it allows for pathologic evaluation and assessment of the surgical margins. In addition, lesions greater than 7–8 cm and those difficult to target with SBRT, are better left for surgical management. [55]

Although traditionally radiation therapy was thought to be immunosuppressive, there is increasing pre-clinical and clinical evidence that high dose, hypofractionated radiation –SBRT-may reverse antitumor immunity via CD8+ T-cells and cellular stress signals. [55] Although uncommon, the abscopal effect (regression in tumors distant to the targeted field of radiation) is an example of recovery of anti-tumor immunity following RT [57]. While the exact mechanism is unknown, it has been proposed that the radiation effect may result in the release of anti-tumor proliferative antigens and cytokines. Matzinger [58] theorized a ‘danger model’, which suggested that the immune system is stimulated by injured tissues rather than ‘non-self’, therefore damaged tissues in irradiated sites may stimulate the immune system. Lee et al. [59]demonstrated in a murine model that hypofractionated, high dose RT may possibly reverse T-cell unresponsiveness to primary and metastatic tumors. More specifically, considering that local RT to a tumor may modify its microenvironment by producing inflammatory cytokines that may increase its immune responsiveness, Lugade et al. demonstrated that IFN-γ (a cytokine), following RT, promotes T cell function [60]. It is thought that a tumor has ongoing crosstalk with the immune system-from one end of a spectrum where the immune system eliminates the tumor, to tumor-immune system equilibrium (a subclinical tumor), until selective pressure from the immune system stimulates the evolution of tumor cells resistant to the immune system (clinically detectable). A way to think of how RT may promote immune recovery is that the irradiated tumor is converted into a ‘vaccine’ that promotes tumor specific T cells to bestow immune memory against non-treated tumors. [57]. Thus, an emerging advantage of SBRT is the possibility that it may be exploited to benefit the immune system.

### Surgery

Historically, most of the surgical data in regards to oligometastatic disease is centered on hepatic resection. [37]. Perioperative mortality related to hepatic resection has decreased from 20% (before 1980) to 1%. Recent improvement in overall survival, following hepatectomy, are likely from improvements in patient selection (shift in definition of resectability to new criteria based on whether a macroscopic and microscopic complete resection of the liver lesion as well as complete resection of any extrahepatic disease), surgical technique, and more effective adjuvant therapy. The use of portal vein embolization and of neoadjuvant chemotherapy have also expanded the population of patients who are eligible for resection. [61] In a recent series of patients having undergone hepatic resection, despite progression on chemotherapy, the 5-yr survival was 53% [51].

Secondary resection remains a worthwhile therapeutic goal; patients brought to resection by systemic therapy enjoy comparable long-term survival to patients who had resectable disease at the time of presentation, and far superior to those receiving palliative systemic chemotherapy [62]. 10–20% of patients that develop colorectal liver metastases present with, or are converted by systemic treatment to, an oligometastatic state defined as metastatic lesions that are limited in number and involving only a single organ. This type of disease is potentially amenable to local therapeutic modalities, of which hepatic resection is the most effective. [51]

### Cryotherapy

Cryotherapy has been utilized in multiple settings for the ablation of metastatic disease [63, 64]. In the largest series published to date, Littrup and colleagues treated a total of 251 oligometastatic tumors from multiple primary cancers in 126 patients [65]. Sites of treatment included retroperitoneal, superficial, intraperitoneal, bone, and head and neck; average diameter of tumors was 3.4 cm. At 11 months average follow-up (range, 0–82 months), a 10% total recurrence rate (26 of 251) was noted; three occurred within the ablation zone, for a local progression rate of 1.2%. The average time to recurrence was 4.9 months, and, at 21 months, the initial ablation zone had reduced in volume by 93% [65].

### Treatment of oligometastases in different cancers

Anecdotal reports of tumors with limited metastases, having undergone treatment to the metastases and had long-term response date back to the 1930's [66]. Several studies, with a variety of endpoints and meeting different levels of evidence criteria, have been performed in a variety of cancers to treat oligometastatic disease. For each clinical study reviewed, we considered the definition of oligometastasis that was used, the therapy given, and sample size. In order to provide an ordinal categorization to assess the strength of the study designs and of the study endpoints, we used the National Cancer Institute Levels of Evidence for Adult and Pediatric Cancer Treatment Studies ranking system to assess each study [67]. In this system, the strength of the studies in descending order is: randomized controlled trials (double-blinded): Level 1i > randomized controlled trials (non-blinded): Level 1ii > non-randomized controlled clinical trials: Level 2 > case series (population-based, consecutive series): Level 3i > case series (consecutive cases (not population-based)): Level 3ii > case series (nonconsecutive cases): Level 3iii. The system ranks the study endpoints in descending order of strength as follows: total mortality: Level A > cause-specific mortality: Level B > quality of life: Level C > event-free survival: Level Di > disease-free survival: Level Dii > progression-free survival: Level Diii > tumor response rate: Level Div.

### Breast cancer

Metastatic breast cancer (MBC) at diagnosis constitutes 3.5–7% of all new breast cancers [68], while oligometastases comprises 1–3% of the total MCB population [50]. Traditionally MBC patients were managed primarily with systemic modalities, with limited local treatments given only for palliative intent.

We searched PubMed using the terms ‘breast oligometastases’, ‘breast oligometastasis’, and ‘oligometastatic breast cancer’; results were as follows, *n* = 20, *n* = 5, and *n* = 51, respectively. We omitted reviews (including special features), studies including mixed primary tumors, case reports, studies not in the English language, studies not focused on oligometastatic breast cancer, and a survey. There were seven clinical studies remaining; oligometastases held six definitions and each study used a different therapy (Table 4).

**Table 4:** Oligometastatic breast cancer

1st Author, year [Ref]	Strength of evidence (study design /endpoint)	Prospective (P) or Retrospective (R)	Sample size	Definition-Oligometastases	Therapy	Endpoint	Conclusion
Kobayashi, 2012 [71]	3ii /A	R	75	1–2organs with met lesions, ≤5 lesions/organ, ≤5cm lesion diameter	+/− CT, then +/− local therapy + CT	10yr OS-59.2%; 20yr OS 34.1%	Prognosis of OMBC superior to that of MBC
Bojko, 2004 [69]	3iii / A	P	48	1 organ with 1-few met lesions	Surgery or RT + CT, then peripheral-blood-stem-cell transplant	MOS-42.2 mths	Combined modality therapy safe in OMBC; promising relapse-free survival
Milano, 2009 [70]	3iii / A	P	40	≤5 met lesions	Curative-intent SBRT	4yr OS-59%; MOS- NR	SBRT may yield prolonged survival + perhaps cure in select OMBC
Mimoto, 2014 [72]	3iii /A	R	14	1–2 organs with met lesions, ≤5 lesions/organ, ≤5cm lesion diameter	Surgery	10yr OS-59.2%, 20yr OS-34.1%; CD44+/CD24–/low tumor cells in 9% OMBC *versus* 73% non-OMBC	In OMBC, low levels of cancer-initiating cells may be associated with better prognosis
Vander Walde, 2012 [73]	3iii /A	R	12	≤3 sites	CT, then peripheral stem cell rescue	3-yr OS- 73%	Therapy was safe
Nieto, 2002 [75]	3iii /A	R	60	Low tumor burden, w met lesion could be either excised en bloc before HDC, or encompassed w a single RT field w curative intent.	CT	MOS- 80 mths; 5-yr OS 62%	Possibly re-evaluate current tenet that early detection MBC is of no benefit
Bourgier, 2010 [74]	3iii /D	R	159	1 met site	RT *versus* RT + surgery	3yr OS- RT- 39% *versus* RT+ surg-57%; equivalent when adjusted for prognostic factors	In sub-analysis, OMBC had better metastatic PFS as compared to patients with >1 met site

Abbreviations: Met(s) = metastasis (es); CT = chemotherapy; yr = year; OS = overall survival; OMBC = oligometastatic breast cancer; MBC = metastatic breast cancer; RT = radiation therapy; MOS = median overall survival; NR = not reached; mths = months; SBRT = stereotactic body radiation therapy; HDC = high dose chemotherapy; PFS = progression free survival

The evidence for treatment of oligometastases in breast cancer is weak based on the study designs. There were two prospective studies [69, 70], however they were both nonconsecutive case series (Level 3iii) as were the remaining studies, with the exception of one consecutive case series (Level 3ii) [71]. There was a conglomerate of therapies given for patients diagnosed with oligometastases using various definitions. The fact that overall survival endpoints were reported in all studies was helpful (Level A, *n* = 7 studies) [69–75], however, they were not always significant — stronger study designs may have shown whether the survival endpoints were significant. Overall survival (OS) was reported at various intervals making comparisons between studies difficult: median (42.2 months) [69], 20-yr (34%) [71, 72], 5-yr (62%) [75], 4-yr (59%) [70] and 3-yr (73% [73] and 39–57%) [74]).

### Lung cancer

Non-small cell lung cancer (NSCLC) is the leading cause of death worldwide with > 50% of patients having metastatic disease at diagnosis [76, 77]. The primary treatment for most patients with metastatic NSCLC is palliative chemotherapy (CT), which results in median survivals of 8–11 months [78]. However, multiple studies have demonstrated a subset of long-term survivors with OMLC [76, 77]. Additionally, curative outcomes were documented in patients with treated adrenal metastases [79].

We searched PubMed using the terms ‘lung oligometastasis’, ‘lung oligometastases’, and ‘oligometastatic lung cancer’; results were as follows, *n* = 14, *n* = 90, *n* = 123, respectively. We omitted reviews, mixed primary tumors, studies not focused on oligometastatic lung cancer, case reports, editorials, studies not in the English language, surveys, commentaries, cost analyses, studies including less than 10 patients, and studies we were unable to retrieve. There were twenty clinical studies remaining (Table 5); oligometastasis held 17 different definitions and each study had a different treatment paradigm.

**Table 5:** Oligometastatic lung cancer

1st Author, Year [Ref]	Strength of evidence-based on study design / endpoint	Prospective (P) or Retrospective (R)	Sample size	Definition-Oligometastases	Therapy	Endpoint	Conclusion
DeRuysscher, 2012 [81]	2 /A	P	39	<5 synchronous mets	Local trt to mets	MOS-13.5 mths. 3yr OS- 17.5%	Subgroup with synchronous OM may benefit from radical trt
Collen, 2014 [80]	2 /A	P	26	≤5 met lesions	SBRT to primary and all mets	MOS-23 mths. 1yr OS- 67%	SBRT acceptable option and results in acceptable PFS
Khan, 2006 [83]	3i /A	R	23	1–2 sites	CT + local-regional therapy	MOS- 20 mths	Subset of pats may benefit from aggressive local, regional, and systemic treatment
Nieder, 2014 [82]	3i /A	R	23	maximum of 3 metastases to 1 organ	‘Active therapy’, irrespective of specific treatment received	MOS- 11.7 mths for OM and 5.6 mths for advanced mets	Prospective studies for this population are warranted
Guerra 2012 [85]	3ii/A	R	78	<5 mets at diagnosis	Definitive CRT to primary + mets	3yr OS-25%	Tumor volume, KPS, + at least 63Gy to primary tumor are associated with improved OS in OM NSCLC
Ashworth, 2014 [84]	3ii /A	R	757	Hx of curative trt to primary and w 1–5 mets treated w surgery, RT or XRT	Controlled primary tumor and locally ablative treatments to all mets	MOS-26 mths, 5yr OS- 29.4%;	Significant OS differences in OM according to type of metastatic presentation and N status
Collaud, 2012 [86]	3iii /A	R	29	Synchronous single organ met	Lung resection and local trt to mets	1yr OS- 65%, 5yr OS- 36%; MOS- 20.5 mths	Multimodality trt including lung resection should be considered in select pats
Congedo, 2012 [87]	3iii /A	R	53	Resected primary with 1–2 met lesions considered to be resectable	Trt with curative intent	5yr OS- 24%, MOS- 19 mths	Surgical trt for selected patients is feasible and safe
Hasselle 2012 [88]	3iii/A	R	25	≤5 mets	Hypofractionated image-guided RT (HIGRT)	MOS- 22.7 mths; 18mth OS- 52.9%	HIGRT for OM NSCLC provides durable control in ≤2 lesions
Ashworth, 2013 [76]	3iii /A	R	2176	1–5 mets	Surgery, SART or SRS	5yr OS-8.3–86%, MOS- range- 5.9–52 mths	Survival times for OM were highly variable, however long-term survivors do exist.
Griffioen, 2013 [89]	3iii /A	R	61	1–3 synchronous mets	Radical trt (Surgery or RT) to primary and mets	MOS- 13.5 mths; 2yr OS- 38%	Radical trt to selected pats can result in favorable 2yr survival
Yano 2013 [90]	3iii/Diii	R	13	Completely resected NSCLC, with post-op recurrence, excluding secondary lung site. 1–3 distant mets, not brain only	Resection or RT of mets *versus* CT of mets	Median PFS resection/RT-20 mths; Median PFS for CT was 5 and 15 mths, respectively	Local therapy is a choice for 1^st^ line treatment in post-op OM recurrence
Yu 2013 [91]	3iii/A	R	18	EGFR-mutant lung cancer previously treated with erlotinib or gefitinib, then progression on EGFR TKI therapy, (<5 sites disease)	RT, RFA, or resection of a site of progressive disease	MOS from local therapy was 41 mths	EGFR-mutant lung cancers q acquired resistance to EGFR TKI therapy are amenable to local therapy to treat OM disease when used in conjunction with continued EGFR inhibition
Endo, 2014 [98]	3iii/A	P	20	single-organ met, or single-organ metachronous met s/p resect path T1–2N0–1 lung cancer	Resection primary tumor and mets	5yr OS-44.7%	Resection of primary tumor and mets had outcomes comparable to stage II patients
Gray, 2014 [92]	3iii /A	R	66	1–4 synchronous brain mets	Aggressive thoracic therapy (ATT) Surgery or CRT *versus* no-ATT	MOS-26.4 mths for ATT *versus* 10.5 mths no-ATT	Aggressive management of thoracic disease in OM NSCLC associated with improved survival
Cheufou, 2014 [93]	3iii /A	R	37	Synchronous single brain met	Resection cerebral mets and primary tumor	2yr OS- 24%	No increased risk of complication or mortality; median survival encouraging
Parikh, 2014 [94]	3iii /A	R	186	≤5 synchronous distant met lesions	Definitive primary therapy	MOS-17 mths for OM *versus* 14 mths for advanced disease; Among OM, MOS- 19 mths for definitive therapy *versus* 16 mths for no definitive therapy	Definitive therapy to primary tumor may provide survival benefit
Sheu, 2014 [95]	3iii/A	R	90	≤3 synchronous mets	CT, then Surgery or RT before disease progression. Then +/−comprehensive local therapy (CLT)	MOS- 22.3 mths; 1yr OS- 75%	CLT associated with improved OS and PFS with matched analysis using propensity score's
Tonnies, 2014 [96]	3iii/A	R	99	Solitary hematogenous metastasis within 3 mths of primary resection	Primary NSCLC curatively resected; then metastasectomy	5yr OS- 38%	Metastasectomy for synchronous OM NSCLC can be performed in selected patients
Ouyang, 2014 [97]	3iii/A	R	95	Not defined	3DRT + CT	3yr OS-15.8%	Radiation dose ≥63Gy and having bone only mets associated with better OS; aggressive thoracic radiation may play a role in improving OS

Abbreviations: Met(s) = metastasis(es); Trt = treatment; MOS = median overall survival; OM = oligometastases; SBRT = stereotactic body radiation therapy; mths = months; PFS = progression free survival; CT = chemotherapy; CRT = chemoradiation therapy; SART = Stereotactic ablative radiation therapy; KPS = Karnofsky performance status; OS = overall survival; NSCLC = non-small cell lung cancer; Hx = history; RT = radiation therapy; XRT = external radiation therapy; N = node; EGFR = epidermal growth factor receptor; TKI = tyrosine kinase inhibitor; RFA = radio-frequency ablation

The evidence for treatment of oligometastases in lung cancer was weak-to-moderate based on the study designs. There were two nonrandomized controlled clinical trials (Level 2) [80, 81], however the sample sizes were small (*n* = 26 and *n* = 39, respectively). There was one large study (*n* = 2176), although it was retrospective and a nonconsecutive case series (Level 3iii) [76]. Of the remaining 17 studies, two were population-based consecutive case series (Level 3i) [82, 83], two were non population–based consecutive case series (Level 3ii) [84, 85], and twelve were nonconsecutive case series (Level 3iii) [76, 86–97], one of which was prospective [98]. Therapies given varied not only among the studies but within most of them as well, for patients with diversely defined oligometastases. Overall survival endpoints (level A) were reported in all but one study [90], however stronger study designs would have shown whether the survival endpoints were significant. OS was reported as MOS (11.7 [82], 17 [94], 20 [83], 26.4 [92], and 41months [91] and at four intervals in the remaining studies: 5-year (29.4% [84], 8.3–86% [76], 44.7% [98], 38% [96], 36% [86], and 24% [87]), 3-year (17.5% [81], 25% [85], and 15.8% [97]), 2-year (24% [93]and 38% [89]), 18-month (52.9%) [88] and 1-year (67% [80]and 75% [95]).

### Melanoma

Approximately 30% of patients with melanoma will develop metastases. The 5-yr survival of stage IV melanoma is about 5%.

**Table 6:** Oligometastatic melanoma

1st Author, Year [Ref]b	Strength of evidence-based on study design / endpoint	Prospective (P) or Retrospective (R)	Sample size	Definition-Oligo metastases	Therapy	Endpoint	Conclusion
Essner, 2004 [99]	3i /A	R	877	1 met	Curative surgery	5yr OS- 29 mths if mets 1 site, 16 mths if mets 2–3 sites, 14 mths if met ≥4 sites. 5yr OS- 17% disease-free if distant mets in <36 mths, 30% if >36 mths	Pats with limited mets should be considered for curative resection
Knisely, 2012 [100]	3iii /A	R	77	Brain mets treated with SRS	SRS to brain mets, then 35% of group received ipilimumab	MOS- 21.3 mths in ipilimumab group *versus* 4.9 mths in no-ipilimumab group. 2yr OS- 47% in ipilimumab group and 19.7% in no-ipilimumab group	Survival of patients with melanoma and brain mets managed with ipilimumab + SRS can exceed expected 4–6 mths

Abbreviations: Met(s) = metastasis(es); OS = overall survival; SRS = stereotactic radiosurgery; MOS = median overall survival

We searched PubMed using the terms ‘melanoma oligometastasis’, ‘melanoma oligometastases’ and ‘oligometastatic melanoma’; results were as follows, *n* = 5, *n* = 5, and *n* = 15, respectively. We omitted reviews, studies including mixed primary tumors, and studies not focused on oligometastatic melanoma. An additional search through the bibliographies of the review papers allowed us to retrieve one additional clinical study for review, therefore we reviewed two clinical studies (Table 6). The two studies differed in their therapies, which included resection [99], and SRS followed by +/− immunotherapy [100], and definitions of oligometastases.

The evidence for treatment of oligometastases in melanoma is weak based the two, retrospective studies conducted. Essner et al. was a population-based consecutive case series (Level 3i), which included a large sample size (*n* = 877), and determined factors prognostic for increased survival [99]. The second study was a nonconsecutive case series (Level 3iii) with a more moderate sample size [100]. The two studies differed in their therapies, definitions of oligometastases, and interval endpoint of OS. Although OS was reported in both studies (Level A), it was reported in different intervals; 2-year (19.7–47%) [100] and 5-year (17–30%) [99].

### Colorectal cancer

Colorectal cancer is the fourth most common cancer diagnosis in the world (around 1.2 million diagnoses each year), and accounts for the second highest number of deaths [37]. Nearly one-fourth of patients with newly diagnosed colorectal cancer (CRC) will present with synchronous liver metastases [101, 102]. Hepatic resection is considered a standard treatment option for metastatic CRC and can result in 10-year survival rates of 20–26%, and potential cure [103–109].

We searched PubMed using the terms ‘colorectal cancer oligometastasis’, ‘colorectal cancer oligometastases’, and ‘oligometastatic colorectal cancer’; results were as follows, *n* = 5, *n* = 24, and *n* = 28, respectively. We omitted reviews, studies including mixed primary tumors, studies not focused on oligometastatic colorectal cancer, case reports, perspectives, pre-clinical reports, and earlier reports if later reports for the same study were available. There were nine studies remaining (Table 7); oligometastasis held 9 different definitions and each study had a different treatment paradigm.

**Table 7:** Oligometastatic colorectal cancer

1st Author, Year [Ref]	Strength of evidence- based on study design / endpoint	Prospective (P) / Retrospective (R)	Sample size	Definition- Oligo metastases	Therapy	Endpoint	Conclusion
Engels, 2012 [104]	2 /A	P	24	≤ 5 mets	Resected primary tumor, and inoperable mets treated with helical tomotherapy	1yr OS- 78%	Helical tomotherapy is an attractive for consolidation of inoperable OM disease after effective chemo
Dellas, 2012 [110]	2/ D	P	9	1–3 mets or local recurrence plus max. 2 mets	CT + 3D-CRT to all met lesions	3/9 pats survived 3.5–4.4 yrs; DLT not documented	3D-CRT to mets feasible in addition to standard CT
Van den Begin, 2014 [113]	3ii/A	R	47	Resected primary + ≤5 mets, liver, lung, LNs	SBRT	1yr OS- 53%	Nature + location of local recurrences demonstrated need for breathing management and dose >75Gy.
Filippi, 2014 [114]	3ii/A	R	40	Resected primary + 1–5 lung mets, max diameter <5cm	3D conformational RT or image guided volumetric modulated arc therapy	5yr OS- 39%	Suggests stereotactic ablative RT is safe + efficacious in CRC with lung oligometastases
Kang, 2010 [117]	3iii /A	R	59	1–4 met lesions confined to 1 organ, largest <7cm, progressive after CT	Met lesions progressed after chemo, then treated SBRT	5yr OS- 29%	Patients generally fare well after SBRT
Salah, 2012 [112]	3iii /A	R	927	Underwent lung metastasectomy	Lung metastasectomy	5yr OS- 54.3%. Prognostic risk groups good-, intermediate-, and high-, had 5yr OS- 68%, 46%, 26%	More studies need to investigate if surgery offers advantage over CT in the poor-risk group
Bae, 2012 [116]	3iii/A	R	41	Met lesions confined to 1 organ	3 fractions SBRT	5yr OS- 38%	SBRT results comparable with Surgery
Salah, 2013 [115]	3iii /A	R	148	Repeat resection of pulmonary mets	Repeat resection of lung met	5yr OS- 52% for 1 lung met resection, 58% 2 lung met resection. >2 lung mets + ≥3 cm risk factors for decreased survival	In selected patients, repeated pulmonary resection offers good survival outcome
Comito, 2014 [111]	3iii/A	P	82	1–3 inoperable mets in 1 organ (Liver or lung)	SBRT	3yr OS- 43%	SBRT safe + feasible treatment for mets not amenable to resection

Abbreviations: Met(s) = metastasis(es); OS = overall survival; CT = chemotherapy; CRT = chemoradiation therapy; DLT = dose-limiting toxicity; SBRT = stereotactic body radiation therapy; LNs = lymph nodes; RT = radiation therapy; CRC = colorectal cancer

The evidence for treatment of oligometastases in colorectal cancer is weak-to-moderate based on the study designs. There were two prospective, nonrandomized controlled clinical trials (Level 2) studies [104, 110], however both with small sample sizes (*n* = 24 and *n* = 9, respectively) and one prospective nonconsecutive case series (Level 3iii) study with a moderate sample size (*n* = 82) [111]. Salah et al. conducted a pooled analysis of 8 studies with a large sample size (*n* = 927) and reported 5-yr survival, however it was retrospective and a nonconsecutive case series (Level 3iii) [112]. The remaining five studies were (non population-based consecutive case series (Level 3ii) [113, 114] and Level 3iii [115–117]. Overall survival endpoints (Level A) were reported in all but one study [110], however stronger study designs would have shown whether the survival endpoints were significant. OS was reported at three intervals: 5-year (52–58%) [115], 54.3% [112], 39% [114], 38% [116] and 29%) [117], 3-year (43%) [111], and 1-year (78%) [104] and 53%) [113].

### Sarcoma

Soft tissue sarcomas are relatively rare with about 10, 000 cases diagnosed each year in the United States. If diagnosed early, the prognosis is excellent, however, in some subtypes (of which there are greater than 50), up to 50% of patients will develop distant metastases and will have a 5-year survival of below 10%. The median overall survival for patients with advanced and metastatic soft tissue sarcoma is about 12 months [118].

We searched PubMed using the terms ‘sarcoma oligometastasis’, ‘sarcoma oligometastases’, and ‘oligometastatic sarcoma’; results were as follows, *n* = 3, *n* = 3, and *n* = 13, respectively. We omitted reviews, studies including mixed primary tumors, studies not focused on oligometastatic sarcoma, and case reports. There were two clinical studies remaining (Table 8); both studies used different definitions of oligometastasis and a different treatment paradigm.

**Table 8:** Oligometastatic sarcoma

1st Author, Year [Ref]	Strength of evidence-based on study design / endpoint	Prospective (P) or Retrospective (R)	Sample size	Definition-Oligometastases	Therapy	Endpoint	Conclusion
Falk, 2014 [119]	3ii /A	R	281	1–5 lesions, any site	164/281 pats received local treatment (surgery, radiofrequency ablation, or RT	MOS- was 45.3 for local treatment group and was12.6 mths for non-local treatment group	Local ablative treatment seemed to improve OS. Surgery yielded most relevant results, alternative approaches were promising
Rhomberg, 2008 [120]	3iii/Diii	R	16	<7 distant mets	Received CT + RT +/− surgery;	Median survival until 1^st^ distant mets- 17 mths in OM sarcoma *versus* 9 months in control group	Razoxane, vindesine + RT feasible in early met soft tissue sarcoma; inhibits development of remote mets in most patients

Abbreviations: RT = radiation therapy; MOS = median overall survival; OS = overall survival; mets = metastases; CT = chemotherapy; OM = oligometastases

The evidence for oligometastases in sarcoma was weak based on the two retrospective studies available for review. Both studies were nonconsecutive case series (Level 3iii). Falk et al. included a moderate sample size (*n* = 281) and MOS was reported (45.3%) (Level A) [119]. The second study was small (*n* = 16) and the endpoint was time to progression (17–months) (Level Diii) [120].

### Renal cell carcinoma

Approximately 25–30% of patients diagnosed with renal cell carcinoma have metastatic disease at initial presentation. Approximately 1/3 with clinically localized primary tumor at diagnosis will eventually develop metastatic disease [121]. Historically patients with metastatic RCC (mRCC) have a poor prognosis, with 5-yr survival rates of ≤ 10%, however prolonged survival has been noted in those with solitary or oligometastatic disease amenable to resection. RCC is often considered resistant to cytotoxic chemoconventional RT and cytokine-based immunotherapy. The lung is the most common site of metastases in mRCC; the second most common is bone. SABR is being suggested as a potential new therapeutic option.

The standard of care in mRCC is systemic therapy; however, in patients with solitary or limited metastases, aggressive local therapies may potentially prolong survival. The literature suggests a survival benefit with surgical metastasectomy, with a reported 5-year survival as high as 45% in those who achieve complete resection. [55]

We searched PubMed using the terms ‘renal cell carcinoma oligometastasis’, ‘renal cell carcinoma oligometastases’, and ‘oligometastatic renal cell carcinoma’; results were as follows, *n* = 1, *n* = 9, and *n* = 3, respectively. We omitted reviews, studies including mixed primary tumors, and studies not focused on oligometastatic renal cell carcinoma. There were three clinical studies remaining (Table 9); each study used a different definition of oligometastases and a different treatment paradigm.

**Table 9:** Oligometastatic Renal Cell Carcinoma

1st Author, Year [Ref]	Strength of evidence-based on study design / endpoints	Prospective (P) or Retrospective (R)	Sample size	Definition-Oligometastases	Therapies	Endpoints	Conclusion
Mickisch, 2001 [125]	1ii /A	P	85	N/A- patients identified as having metastatic RCC	Surgery + interferon OR interferon only	TTP (5 *versus* 3 mths) + MOS (17 *versus* 7 mths) in Surgery + interferon *versus* interferon only	Radical nephrectomy before interferon-based immunotherapy may delay TTP and improve survival in mRCC
Flanigan, 2001 [126]	1ii /A	P	241	N/A- patients identified as having metastatic RCC	Surgery followed by interferon OR interferon alone	Surgery followed by interferon MOS- 11.1 mths *versus* interferon alone MOS-8.1 mths	Nephrectomy followed by interferon had longer survival
Bang, 2012 [124]	3iii/A	R	27	Localized soft tissue mass <7cm + ≤5 lesions in 1 organ	Cryoablation	5yr OS- 27%	Multisite cryoablation of OM RCC associated with low morbidity and low recurrence with apparent increased OS
Ranck, 2013 [122]	3ii/A	R	18	Limited metastatic disease	SBRT: 3 fractions or 10 fractions	2yr OS- 85%	SBRT produces promising lesion control with minimal toxicity
Thibault, 2014 [123]	3iii/A	R	13	<5 spinal mets	SBRT	1yr OS- 83.9% in OM RCC (n = 13) versus 52.5% in non-OM RCC (n = 24)	Multivariate analysis identified OM RCC as a prognostic factor for survival. OM RCC may benefit the most from aggressive local therapy

Abbreviations: TTP = time to progression; mRCC = metastatic renal cell carcinoma; MOS = median overall survival; OM = oligometastatic; OS = overall survival; SBRT = stereotactic body radiation therapy; mets = metastases

The evidence for treatment of oligometastases in renal cell carcinoma is weak based on the study designs. There were three retrospective studies with smaller sample sizes; a non population-based consecutive case series (Level 3ii) [122] and two nonconsecutive case series (Level 3iii) [123, 124]. Overall survival endpoints (Level A) were reported in at three intervals: 5-year (27%) [124], 2-year (86%) [122], and 1-year (83.9%) [123].

Additional searching through the bibliographies of other selected papers allowed us to retrieve two clinical studies that investigated the effects of removal of the primary tumor in patients with metastatic renal cell carcinoma (mRCC) (Table 9) [125, 126]. The evidence for removal of the primary tumor in mRCC was strong; both studies were nonblinded randomized controlled clinical trials (Level 1ii), had moderate sample sizes, and reported median OS (17 *versus* 7 months, [125] and 11.1 *versus* 8.1 months [126]) (Level A). The therapies given in both studies were similar: nephrectomy + interferon *versus* interferon only [125] and nephrectomy was followed by interferon *versus* interferon only [126]. The evidence for oligometastases in RCC was not assessed in these studies. However, they were important studies owing to strong design, with comparable treatments and endpoints, and most importantly they demonstrated that removal of the primary tumor in metastatic disease increased survival.

### Prostate cancer

There is a body of evidence, contrary to historical clinical practice, that treating men with metastatic prostate cancer to the lymph nodes at the time of diagnosis with surgery or radiation therapy results in increased long-term survival (Table 10), [127–131] and Table 11 [101, 132]. Historically, the ideal patient to be cured by radical prostatectomy (RP) was one with organ-confined cancer. At that time, the morbidity of RP was substantial; therefore the surgery was generally only offered to those where the probability of cure was high. More recent data, however, show that RP may provide a survival benefit-albeit not a cure — to men with metastatic prostate cancer. Engel et al. [101] conducted a retrospective study (*n* = 938) and found that in men with prostate cancer and positive lymph nodes, treated with +/− RP, that the 5- and 10-yr survival rates and the prostate cancer specific survival rates, were better in men who had undergone RP. Cadeddu et al [132] conducted a retrospective study (*n* = 38) and found that in men with prostate cancer and positive lymph nodes, treated with LND +/− RP, that the 5- and 10-yr prostate cancer specific survival was better in men who had undergone LND + RP. Ost et al (2014) conducted a systemic review of the literature of metastasis-directed therapy of regional and distant recurrences after curative treatment for PCa (prostate cancer). They found that salvage LND and RT appear to be safe in treatments for OM PCa recurrence. [133] Culp et al. [134] studied the impact of survival of definitive treatment of the prostate in men diagnosed with metastatic PCa. Using the SEER database, he reviewed 8185 men treated with NSR (no surgery no radiation), brachytherapy, or RT. The 5-year OS and disease specific survival (DSS) was significantly higher in men with metastases having undergone RT.

**Table 10:** Prostate cancer (staged T1c-T3b [198])

1st Author, Year [Ref]	Study population	Strength of evidence-based on study design / endpoint	Prospective (P) / Retrospective (R)	Sample size	Therapy	Endpoints	Conclusion
Widmark (2009) [127]	Locally advanced	1ii /A	P	875	Primary: HT +/− RT	10yr PCa specific mortality in ET + RT group and the ET alone group was 11.9% and 23.9%. 10yr PSA recurrence higher in the ET alone group (74.7% vs. 29.5%	Addition of RT to ET, halved 10-yr PCa-specific mortality and decreased overall mortality in locally advanced PCa
Vickers (2012) [128]	T1–T2	1ii /B	P	695	Primary: RP	RP beneficial to Gleason 8, or Gleason 7, stage 2	Younger men w more aggressive disease had larger reduction in risk of PCa death
Zelefsky (2010) [129]	Clinically localized: T1c-T3b	3iii /B	R	2380	Primary: RP or RT to prostate	8-year probability of freedom from met progression was 97% for RP and 93% for EBRT	RP with higher risk disease, had lower risk of met progression and PCa specific death
Pierorazio (2013)[130]	Up to clinically localized	3iii /B	R	842	Primary + LNs: RP+PLND	PSA ≥20 and perineural invasion at biopsy increased likelihood of unfavorable, high-grade disease.	High-Gleason PCa not uniformly associated with poor outcomes after RP, but unfavorable (pT3b/N1) disease fared poorly
Shao (2014) [131]	Localized	3iii /B	R	916	Primary: RP or RT	RP had longer PCaSS as compared to primary RT	Results add to the growing evidence that controlling the primary site may be important in patient with met cancer

Abbreviations: HT = hormone therapy; RT = radiation therapy; PCa = prostate cancer; ET = endocrine therapy; RP = radical prostatectomy; EBRT = external beam radiation therapy; LNs = lymph nodes; PLND = positive lymph node dissection; PCaSS = prostate cancer specific survival; met = metastatic

**Table 11:** Oligometastatic prostate cancer

1st Author, Year [Ref]	Strength of evidence-based on study design / endpoints	Prospective (P) / Retrospective (R)	Sample size	Definition-Oligometa stases	Therapy	Endpoints	Conclusion
James, 2014 [137]	1ii /A	P	917	N/A – newly diagnosed M1	LT ADT	FFS- 11 mths. 2yr FFS- 29%. MOS- 42 mth. 2yr OS- 72%.	Survival disappointing in M1 disease started only on LT ADT, despite active treatments available at first ADT failure. Spend most of their time in CR relapse.
Singh, 2004 [149]	3iii/A	R	30	≤5 met lesions	External RT	5- and 10yr OS- 73% and 36% in OM, as compared to 45% and 18% in those with > 5 met lesions	Findings suggest early detection and aggressive treatment is worth testing to improve long-term survival
Engel, 2010 [101]	3iii /A	R	938	+LNs	+/− RP	5yr- and 10yr OS- 84% and 64% with completed RP, and 60% and 28%, with aborted RP. PCa-specific survival at 5- and 10-yrs- 95% and 86%, with completed RP and was 70% and 40%, with aborted RP	Abandonment of RP in men with positive LNs may not be appropriate
Tabata, 2012 [148]	3iii/A	R	35	<6 bone mets on bone scan, each site less than 50% the size of a vertebral body	RT	3yr OS- 77%; 14/16 (87%) of pats who had pain were improved 1 mth after RT; median duration of pain control 12 month	RT for bone OM in PCa was effective for long-term pain relief
Schick, 2013 [141]	3iii /A	R	50	1–4 mets, synchronous or metachronous	Mets- ADT and HDRT	3yr biochemical relapse-free survival (bRFS), clinical failure-free survival, and OS- 54.5, 58.6, and 92%	OM may be treated w short ADT and HDRT to the met regions. High dose improves bRFS. May prolong failure-free interval between 2 consecutive ADT courses.
Ponti, 2014 [143]	3iii/A	R	16	Distant relapse in a limited number of regions, ≤5 mets	SBRT +/− HT	Local control, biochemical PFS, OS, toxicity. OS at 29 mths 95% Distant relapse in a limited number of regions, ≤5 mets	SBRT safe, effective, minimally invasive in limited LN recurrence in OMPCa
Jereczek-Fossa, 2014 [147]	3iii/A	R	69	Single abdominal LN recurrence	SBRT	3-yr in-field PFS, PFS, OS- 64%, 11.7%, and 50%	SBRT is feasible for single abdominal LN recurrence, offering excellent in-field tumor control.
Cadeddu, 1997 [132]	3iii /B	R	38	+LN: pelvic lymph adenopathy	PLND +/− RP	PCa-specific survival at 5- and 10-yrs- (93% and 56% in the PLND/RP group and 58% and 34% in the PLND group	RP, as compared to conservative therapy, may prolong survival
Ahmed, 2013 [145]	3iii/B	R	17	≤5 met lesions	SBRT	Local control-100% at 6mo; cancer specific survival (CSS)-6- and 12mo-100%; freedom from distant progression (FFDP)- 6- and 12mo- 74%, 40%	Excellent LC with SBRT for OM PCa; over 50% patients achieved undetectable PSA after SBRT
Ost, 2014 [142]	3iii /B	R	80	Metachronous mets	Mets- ADT, AS, or MDT	Median PCSS- 6.6 yrs.	Longer PSA DT, involvement of nodes or axial skeleton and lower # mets assoc w improved PCSS.
Decaestecker, 2014 [144]	3iii/B	R	50	≤3 metachronous asymptomatic mets	SBRT (2 RT schedules used) +/− HT	Median PFS- 19mo; median ADT-FS- 25 month; 2-, 5yr PCSS-96%, 90%	Repeated SBRT for OM PCa postpones palliative ADT
Berkovic, 2013 [146]	3iii/Di	R	24	Biochemical recurrence after curative treatment to primary (RP, RT, or both), then ≤3 synchronous asymptomatic mets	SBRT	Androgen deprivation therapy-free survival (ADT-FS)- 1-, 2yr-82%, 54%; clinical progression free survival- 1-, 2yr- 72% and 42%	Repeated salvage SBRT feasible, well tolerated, and defers palliative ADT with a median 38mo in OMPca
Ost, 2014 [133]	3iii/Diii	R	450	Metachronous mets with controlled primary, + underwent MDT for recurrent PCa	RT or LND	About 50% PFS at 1–3 yrs post-MDT	MDT promising approach for OM PCa recurrence but low level of evidence

Abbreviations: M1 = distant metastases; LT ADT = long-term androgen deprivation therapy; FFS = failure-free survival; OS = overall survival; CR = castrate resistance; met = metastasis; RT = radiation therapy; OM = oligometastasis; LNs = lymph nodes; RP = radical prostatectomy; PCa = prostate cancer; HDRT = high dose radiation therapy; ADT = androgen deprivation therapy; SBRT = stereotactic body radiation therapy; HT = hormone therapy; PFS = progression free survival; LN = lymph node; OM PCa = oligometastatic prostate cancer; PLND = positive lymph node dissection; LC = local control; PSA = prostate specific antigen; PCSS = prostate cancer specific survival; AS = active surveillance; MDT = metastasis directed therapy; DT = doubling time; ADT-FS = androgen deprivation free survival; LND = lymph node dissection; PFS = progression free survival

Zapatero et al., reported on a study of men with intermediate and high risk localized prostate cancer (*n* = 362), treated with RT + long term ADT *versus* RT + short term ADT, and found that long term ADT was superior in 5-year biochemical disease free survival, metastasis free survival, and OS [135].

The standard of care treatment for metastatic prostate cancer is androgen deprivation therapy (ADT) [136]. Men with newly diagnosed metastatic prostate cancer, entered into the control arm of the Systemic Therapy Multi-Arm Randomized Controlled Trial (STAMPEDE) and treated with ≥ 2 years of ADT alone, had a 2-year OS of 72% [137]. The response rate for primary hormonal therapy for men with metastatic prostate cancer exceeds 80% and the median duration of response is approximately 18–24 months [138]. In men with prostate cancer with limited metastases, radical prostatectomy may be advantageous in that the primary tumor and its ability to continuously metastasize, to secrete tumor promoting growth factors and immunosuppressive cytokines, and to generate bulk-related morbidity, is removed. Heidenreich et al. recently evaluated survival outcomes following radical prostatectomy (RP) in men with low volume metastatic prostate cancer. RP led to improved progression-free survival, time to castrate resistance and overall survival, as compared to a cohort treated with androgen deprivation therapy alone. [139] Moreover, Abdollah et al. found that in men with pN1 prostate cancer, treated with RP and extended lymph node dissection, adding adjuvant radiotherapy improved cancer-specific mortality [140].

We searched PubMed using the terms ‘prostate cancer oligometastasis’, ‘prostate cancer oligometastases’, and ‘oligometastatic prostate cancer’; results were as follows, *n* = 3, *n* = 19, and *n* = 22, respectively. We omitted reviews, studies including mixed primary tumors, studies not focused on oligometastatic prostate cancer, case reports, editorials/commentaries, studies that we were not able retrieve, and studies with no results reported. There were ten clinical studies remaining (Table 11); oligometastasis was defined differently in each study and there were 7 different treatment paradigms.

The evidence for treatment of oligometastases in prostate cancer is weak based on the study designs being all nonconsecutive case series (Level 3iii) [101, 132, 133, 141–149], although Ost el al. included a large sample (450 men) [133]. Overall survival endpoints (Level A) were reported in seven studies: 10-year (36% [149] and 64% [101]), 3-year (50% [147], 77% [148], and 92% [141]), 29-month (95%) [143], and 2-year (72%) [137].

Currently there are two studies, not yet completed, for which we are anxiously awaiting the results. Decaestecker et al. are conducting the first randomized, phase 2 trial on men with an oligometastatic recurrence to assess the impact of metastases-directed therapy - *versus* active surveillance - on the start of palliative ADT [150]. Attard et al., as part of the ongoing STAMPEDE trial, describes the addition of a new treatment arm (enzalutamide, abiraterone and prednisone with ADT) for men with newly diagnosed M1 disease [151].

### Current trials of oligometastatic disease

We reviewed clinicaltrials.gov to gauge the ongoing studies for oligometastatic disease. Multiple trials are underway to determine if the treatment of oligometastatic disease is beneficial in cancer as reported on clinicaltrials.gov [152] (see Table 12).

**Table 12:** Current studies for oligometastatic disease (ClinicalTrials.gov (CTG) as of 2/1/2015)

CTG NCT# / site	Condition	Name	Purpose	Algorithm	Primary Outcome/Endpoint
01859221 University Florida [162]	Prostate oligometastases	Radiotherapy for OMPC	Phase II study to evaluate outcomes of patients treated with Stereotactic radiation therapy for OMPC	RT	Improved PFS over historic controls.
01777802 Mayo Clinic [163]	Prostate oligometastases	Monitoring Anti-Prostate Cancer Immunity Following SBRT	Determine if SBRT conditions solid tumors to be favorable to the initiation of robust antitumoral immune responses	Observation following SBRT	Induction of anti-prostate cancer immunity
02020070 MSKCC [170]	Prostate oligometastases	Ipilimumab, degarelix, + RP in castrate sensitive PC or ipilimumab + degarelix in biochemical recurrent castrate sensitive PC after RP	Assess safety + efficacy of combining HT + immunotherapy in non-castrate resistant PC. Cohort 1: ipilimumab + degarelix pre- and post- RP in newly diagnose OM castrate-sensitive disease. Cohort 2: post definitive local therapy with RP, but with biochemical recurrence	IT, LHRH antagonist + surgery -OR- IT, LHRH antagonist	Undetectable PSA at 12- and 20-mths with non-castrate testosterone.
02264379 Technische U Dresden [165]	Prostate oligometastases	Percutaneous high-dose RT in OMPC	Evaluate outcomes of patients treated with high-dose radiation using either hypofractionated or normofractionated RT; to establish efficacy + safety	HDRT Hypofraction -Or- Normofraction	Toxicity
01558427 UHosp, Ghent [169]	Prostate oligometastases	Salvage treatment of active clinical surveillance for OMPC: PhII RCT	To determine if salvage treatment of OMPC with either surgery or RT might postpone the start of ADT	Surgery -Or- RT, Which delays ADT?	ADT-free survival
02192788 Hospital Provincial de Castellon [164]	Prostate oligometastases	PhII study SBRT as treatment for OMPC	Evaluate effect SBRT for OMPC, regardless of basal treatment received	SBRT	# patients without progression of PC treated by SBRT
02274779 Institut cancerologie de l'Ouest [166]	Prostate oligometastases	PHII trial salvage RT + HT in OM pelvic node relapses of PC	Assess BC or clinical relapse-free survival at 2yrs of PC with 1–5 OM treated with concomitant HDCRT + HT	RT + HT	BC or clinical relapse-free survival at 2yrs
00544830 Institute CoHMCNC [167]	Prostate oligometastases	IMRT in treating patients undergoing ADT for mPC	Assess how well IMRT works in pats undergoing ADT for mPC	HT + RT	Time to PSA relapse
00544830 City of Hope Medical Center [168]	Prostate oligometastases	IMRT in Patients Undergoing ADT for Metastatic Prostate Cancer	Evaluate intensity-modulated radiation therapy works in treating patients undergoing androgen deprivation therapy for metastatic prostate cancer	ADT + IMRT	Time to PSA relapse
01728779 Sidney Kimmel Comprehensive Cancer center [180]	Oligometastases to lung, liver, bone	Stereotactic Body Radiation With Nelfinavir for Oligometastases	Evaluate efficacy radiosensitizer nelfinavir used concurrently with SBRT	Nelfinavir + SBRT	PFS at 6months
01345539 U Pittsburgh [181]	Oligometastatic disease	Radiosurgery for OM Disease at Initial Presentation	Evaluate feasibility of radiosurgery for all metastatic sites in OM	SRS	Feasibility SRS/SBRT in OM at initial presentation
02076477 Sichuan Cancer Hospital + Research Institute [158]	OM stage IV NSCLC	The Optimal Intervention Time of Radiotherapy for OM Stage IV NSCLC	Evaluates optimal time for RT for OM stage IV lung cancer	RT	Short-term effects (response rate using RECIST)
01796288 Wu Jieping Medical Foundation [159]	OM NSCLC	Radiotherapy in Oligometastatic Non-squamous NSCLC With Clinical Benefits From 2^nd^ Line Erlotinib	Evaluate RT in combination with erlotinib in OM NSCLC	Erlotinib +/− RT	PFS
01345552 >U Pittsburgh [182]	Recurrent OM disease	Radiosurgery for Patients Recurrent OM Disease	Evaluate feasibility of SRS in recurrent OM disease	SRS	Being able to complete accrual to study
01565837 Comprehensive Cancer Centers Nebraska [177]	OM Melanoma	Concurrent Ipilimumab and Stereotactic Ablative Radiation Therapy (SART) for OM Unresectable Melanoma	Evaluate if SART + ipilimumab will improve survival in OM melanoma	Ipilimumab + SART	Safety and tolerability
02316002 U Pennsylvania [160]	OM NSCLC	Phase II Study of Pembrolizumab After Curative Intent Treatment for OM NSCLC	Evaluate how well pembrolizumab works in previously treated OM NSCLC	Pembrolizumab	PFS
01646034 Netherlands Cancer Institute [171]	OM Breast cancer	High Dose CT in OM Homologous Recombination Deficient Breast Cancer	Studies effect of high-dose alkylating CT *versus* standard CT in OM breast cancer with recombination deficiency	Carboplatin, thiotepa, and cyclophosphamide *versus* docetaxel, doxorubicin, cyclophosphamide, carboplatin, paclitaxel, gemcitabine	Event free survival
02228356 Universitair Zeikenhuis Brussel [183]	Neoplasm metastasis	Non-interventional Observational Study of SBRT for OM Cancer	Investigate if respiration control and improved technique improve local control	SBRT	1-year local control
01725165 M.D. Anderson Cancer Center [161]	OM Lung cancer	OM Disease	Learn if surgery or radiation after CT help control NSCLC	+/− local consolidation therapy	PFS
02303366 Peter MacCallum Cancer Center, Australia [172]	OM breast cancer	Pilot Study SABR for OM Breast Neoplasia in Combination With the Anti-PD-1 Antibody MK-3475	Assess safety + feasibility of MK-3475 + SABR for definitive treatment of OM breast cancer	SABR + MK-3475	# of patients who complete treatment; safety
01759238 Universitatsklinikum Hamburg-Eppendorf [193]	OM CRC metastases	Chemoradiotherapy for Patients With Oligometastatic Colorectal Cancer	Evaluate role of CRT with capecitabine and bevacizumab in OM patients who are neither progressive nor resectable after CT	Capecitabine, bevacizumab and RT	PFS
01282450 Maastricht Radiation Oncology [157]	Stage IV OM NSCLC	Concurrent and Non-concurrent CRT or RT Alone for OM Stage IV NSCLC	Aim is to improve long term survival in OM NSCLC	RT	OS Note: Study completed
02086721 Maastricht Radiation Oncology [188]	Solid tumors	Assess Toxicity of Immunocytokine L19-IL2 After SABR OM Solid Tumour	Hypothesis is that the immunocytokine L19-IL2 and RT will synergize to improve OS in OM solid tumors	L19-IL2 + RT	Toxicity
01965223 Trans-Tasman Radiation Oncolog Group [189]	Cancer metastases to lung	Randomized Phase II Study SABR Metastases to the Lung	Determine safety of SABR *versus* SRS for OM to lung	Multi-fraction SABR and single fraction SABR	Toxicity
02107755 Ohio State U Comprehensive Cancer Center [178]	Metastatic melanoma	Stereotactic RT and Ipilimumab in Metastatic Melanoma	Determine if stereotactic radiosurgery + ipilimumab kills more tumor cells by causing additional melanoma antigens to be presented to immune system,	Ipilimumab + stereotactic radiosurgery	PFS
01446744 Lawson Health Research institute [190]	Metastatic tumors	SABR for Comprehensive Treatment of OM Tumors	Compare SABR with CT and conventional RT to assess impact on OS and Q of L	SABR versus palliative RT	OS
02264886 Washington U School of Medicine [191]	Central thorax cancer, liver cancer, or non-liver abdominal cancer	Adaptive MRI-Guided SBRT for Unresectable Primary or OM Central Thorax and Abdominal Malignancies	Assess feasibility of RT using MRI-guided adaptive technique (day by day re-planning while patient is receiving treatment)	MRI guided SBRT	Feasibility of MRI-guided SBRT (treatment can be delivered in <80 minutes for >75% of patients)
01761929 Princess Margaret Hospital, Canada [184]	OM solid tumors	5 Fraction SBRT for OM Regimen, for Extra-Cranial OM	Monitor side effects and outcomes from higher doses of RT, while limiting dose to normal tissues (using a 5 day schedule)	RT in 5 fractions	1 year PFS at index site
02170181 U of Texas SW Medical Center [185]	Cancer and receiving RT	Prospective Clinical Registry for OM Disease, Consolidation Therapy, Debulking Prior to CT, or Re-Irradiation	Determine trends in patterns of care and outcomes for refinement and justification of this treatment	SBRT	Patterns of care
02089100 Gustave Roussy, Cancer Campus, Grand Paris [173]	Breast cancer	Trial of Superiority of SBRT in Breast Cancer	Prospectively study role of metastases SBRT with curative intent in de novo OM disease	SBRT *versus* placebo	PFS
01898962 Rocky Mountain Cancer Centers [186]	Stage IV or recurrent carcinoma or sarcoma	Definitive Therapy for OM Solid Malignancies	Aggressive treatment to clinically active sites of disease (alone or + systemic therapy) may improve survival	Definitive local treatment (surgery, RT, radioembolization)	OS and disease-specific survival
00463060 Mount Sinai School of Medicine [187]	Metastatic cancer	Sutent and Radiation as Treatment for Limited Extent Metastatic Cancer	Combine sutent and RT to determine safety and best method of combining the treatments	Sutent + RT	Survival
02231775 M.D. Anderson Cancer Center [179]	Stage IV melanoma	Combi-Neo Study for Stage IV Melanoma	Compare dabrafenib + trametinib before surgery versus surgery alone	Surgery +/−dabrafenib +/−, trametinib	1-year relapse-free survival
01781741 Roswell Park cancer Institute [154]	Stage III-IV NSCLC	SBRT After Surgery in Stage III-IV Non-small Cell Lung Cancer	Evaluate SBRT post lymphadenectomy	Therapeutic lymphadenectomy, SBRT	Toxicity
00776100 North Central Cancer Treatment Group/NCI [156]	Stage IV NSCLC	RT or Observation After CT in Stage IV NSCLC	Randomized Phase II trial studying how well RT works compared to CT in Stage IV NSCLC	RT versus observation	OS Note: Study completed
00182793 City of Hope Medical Center/NCI [174]	Breast cancer	Combination CT +/− Trastuzumab Followed By an Autologous Stem Cell Transplant (SCT) and RT in Stage III or IV Breast Cancer	Evaluate combination CT +/− trastuzumab, then autologous SCT and RT	CT+/− Trastuzumab, then SCT + RT	TTP, OS, 5-year response rate, feasibility Note: study completed
01941654 Chinese University of Hong Kong [153]	NSCLC activating EGFR mutation	ATOM_local Ablative Therapy	Efficacy of local ablative therapy in NSCLC with activating EGFR mutation with active OM disease after 1^st^ line TKI EGFR	Local ablative RT	PFS at 1-year
01185639 Comprehensive Cancer Center Wake Forest U [155]	NSCLC	SBRT in Metastatic NSCLC	Evaluate feasibility, safety, and efficacy of SBRT after 4cycles 1^st^ line CT	SBRT	PFS
01763970 Sidney Kimmel Comprehensive Cancer Center [194]	Pediatric sarcoma	SBRT for Pediatric Sarcomas	Evaluate efficacy of SBRT (5 fractions) in pediatric sarcoma	SBRT	SBRT efficacy
01875666 UNC Lineberger Comprehensive Cancer Center [175]	Breast neoplasm	Defining the HER2 Positive (+) Breast Cancer Kinome Response to Trastuzumab (T), Pertuzumab (P), Combination Trastuzumab +Pertuzumab (T+P), or Combination Trastuzumab + Lapatinib (T+L)	Evaluate kinome response in Stage I-IV HER2+ scheduled to undergo definitive therapy for OM disease	Randomized to T, P, T+P, or T+L	Difference in kinome activation pre- and post-treatment
01706432 U of Chicago / NCI [176]	Stage IV breast cancer	Hypofractionated IGRT in Stage IV Breast Cancer	Studies hypofractionated IGRT in stage IV breast cancer	Hypofractionated RT	Feasibility of correlating number of circulating tumor cells with TTP in metastatic breast cancer
01347333 St. John's Mercy Research Institute, St. Louis [192]	Liver metastases, hepatocellular carcinoma, intrahepatic cholangiocarcinoma	SBRT for Liver Tumors	Evaluate local control rate of SBRT to liver tumors	SBRT	Local tumor recurrence rate

Abbreviations: OMPC = oligometastatic prostate cancer; RT = radiation therapy; PFS = progression free survival; SBRT = stereotactic body radiation therapy; RP = retropublic prostatectomy; PC = prostate cancer; HT = hormone therapy; OM = oligometastatic; IT = immunotherapy; LHRH = luteinizing hormone-releasing hormone; PSA = prostate specific antigen; HD = high dose; RCT = randomized controlled trial; ADT = androgen deprivation therapy; BC = biochemical; IMRT = image guided radiation therapy; PFS = progression free survival; SRS = stereotactic radiosurgery; CRT = chemoradiation therapy; RECIST = response evaluation criteria in solid tumors; NSCLC = non-small cell lung cancer; CT = chemotherapy; OS = overall survival; SABR = stereotactic ablative radiation therapy; MRI = magnetic resonance imaging; SCT = stem cell transplant; TTP = time to progression; ATOM = ablative therapy oligometastases; EGFR = epidermal growth factor receptor; Q of L = Quality of Life

Nine studies are in progress for oligometastatic NSCLC, with therapies ranging from SBRT [153–155], to RT [156, 157], to chemoradiation therapy [158], to targeted therapy +/− RT [159], to immunotherapy [160], to any local therapy [161]. For men with oligometastatic prostate cancer, nine trials are underway investigating a variety of therapies including single agent RT [162–165], RT in combination with HT [166, 168], salvage treatment (surgical or RT) of metastases [169], and combination of HT + immunotherapy [170]. For women with oligometastatic breast cancer, six studies are currently open investigating several therapies including various chemotherapy regimens [171], SABR + antibodies [172], SBRT [173], chemotherapy + antibodies + RT [174], targeted therapies [175] and RT [176]. Three studies are studying oligometastatic melanoma, two are investigating immunotherapy + SABT/SRS [177, 178] and the third study is investigating chemotherapy with and without surgery [179]. There are thirteen studies enrolling patients with oligometastases without a specific primary tumor [180–192], employing a variety of treatment options. Finally, there is one study each for oligometastatic colorectal cancer using chemoradiation [193] and for oligometastatic pediatric sarcoma using SBRT [194].

## CONCLUSIONS

Hellman proposed that tumors early in the chain of progression may give rise to metastases limited in number and location due to an undeveloped metastatic competence of the cancer “seeds” as well as the restrictive nature of the host tissue at some metastatic sites. The rationales of discrete steps of metastases [11, 12] and the hallmarks of cancer [16, 17] have both provided a biologic framework to support the oligometastatic state. Molecular diagnostics have not yet arrived to the point to determine where an individual metastatic deposit is within the continuum of malignancy [32–44, 45], preclinical models are beginning to lay the groundwork of the oligometastatic state [19–31].

While Hellman posited that oligometastasis may be underrecognized due to limitations in imaging diagnostics, imaging has advanced to such a degree that we are now in a yin-yang position such that, 1) imaging often likely demonstrates very early findings not likely to manifest into disease and, 2) imaging demonstrates very early disease that is destined to become widespread, which cannot be distinguished from oligometastases.

Hellman described potential cure as an attractive upshot of treating an oligometastatic state, but prolonged survival has been added as a potential benefit. There is an increasing shift toward individualized, multidisciplinary management of OM disease [34] because it is difficult to conduct randomized controlled trials in oligometastatic patients due to the variety of presentations. While other trial designs may be more realistic, such as N of 1 studies, tissue-based approaches, propensity matched analyses and adaptive design methods [55], Hellman originally endorsed that the ability to identify the oligometastatic state would profit from careful review of past experience, i.e. re-analysis of clinical data found in archival materials to arbitrate when, during the evolution of individual tumors, the oligometastatic state can be found.
